# A Review of the Modern Treatment of Gonorrhea

**Published:** 1908-01

**Authors:** C. W. Canan

**Affiliations:** Orkney Springs, Virginia


### A REVIEW OF THE MODERN TREATMENT OF GONORRHEA.

By C. W. Canan, M. D., B. S., Orkney Springs, Va.

---

The subject under consideration is an old one, but we believe from recent observations that we have found a line of treatment that will give more than ordinary results heretofore accomplished. The acute cases of gonorrhea, as a rule, yield readily to treatment if the patient will co-operate with the physician. On the other hand, the chronic cases, or secondary affections, are the ones that give the physician as well as the patient no end of trouble. Then again, a patient suffering from acute infection is not often so reckless as to spread the disease, but it is while under its secondary influence, when he believes himself cured, that he is most likely to communicate it to those he has marital relations with, whether prostitute or wife. Of all the diseases, I know of none in which the cure has been so hard to effect parmanently, as that of chronic gonorrhea and its many sequelae. It is only within the last few years that we have been able to discover the great damage done to the various organs and functions remote from their local site of action. In fact, we are almost forced to believe that a patient who has had a case of gonorrhea and has let it run into a chronic form, will never be entirely free from the inroads made upon his system.

In treating chronic blennorrhagia, or chronic gonorrhea, there are several things essential to be known before the physician can intelligently treat the patient with the hope of producing a permanent cure. When the patient presents himself at the office with a case of chronic blennorrhagia, the physician should first ascertain definitely the presence of gonococci; he may not be able to do this the first trial, but he should not stop until he satisfies himself of their presence. This being done, the next step is to find their habitat, or that part of the urethral tract in which they live, multiply and keep up their operations. These two discoveries should always be made before the treatment is decided upon. Now the object of the treatment should be difinitely decided upon and divides itself into three things.

First, to destroy the gonococcus completely; second, to suppress as far as possible secondary infections from other microbic invasion, which are often found engrafted on an old gonorrhea and continue after the gonococci themselves have disappeared; third, to take measures that these do not return, or are kept in abeyance, and to treat the urethral and bladder symptoms according to the lesions left behind. So far, so good. Before describing the treatment, we want to caution those who expect to use it, for we believe that epididymitis and other complications are often the result of badly applied treatment, or that of maldirected instrumentation. The urethra must first be rid of its gonococcus as much as possible before the application of the treatment. Our method is as follows; first, massage the prostatic and post urethra as thoroughly as possible to break up the nests or pockets of gonococci and cause the folicles to empty themselves; second, the patient is then directed to urinate as freely as possible, the entire urethral canal is then thoroughly irrigated with some warm, mild antiseptic solution; third, pass the largest catheter possible to stretch out all folds and pockets of mucous membrane that might have escaped the massaging. Then one quart of warm solution of permanganate of potash should be slowly injected into the bladder; the catheter is then withdrawn and the patient directed to void the solution at once; fourth, if necessary to open and scrape fistulous tracts, the laying open of folicles at the meatus, or of follicles about the frenum. Under these four divisions of treatment, we believe that ninety per cent of patients can be cured if the treatment is carried out intelligently. These irrigations should be made daily to the urethra (of some warm, mild antiseptic solution) for a period of from eight to ten days, then an examination should be made to discover the presence of gonococcus. If still present, the same process should be followed as at first and the solution of permanganate made stronger and applied. This is seldom necessary for in the majority of cases, the gonococcus disappear after the first seance and if this is followed by proper treatment for the secondary infections, the case will go on to recovery. To save the patient time, and the physician both time and trouble, the presence of gonococcus can be determined by one of the simpler methods; by having the patient take copious draughts of beer thereby causing a reaction which will reveal their presence. Now in reference to the best remedies to use in those daily injections or irrigations, the writer has found that mercury bichloride1:15000 or 1:10000 in warm solution one of the most effective. Before these injections are made, the glans. prepuce and meatus should be carefully cleansed with the same solution. Nitrate of silver 1:2000 in some cases does well. The same is true of boracic acid in solution. If ulcers or diseased follicles are found along the urethral canal, they should be properly treated according to indications. The same is true of all complications. It is hardly necessary for us to caution the reader in reference to absolute cleanliness in every step of this work. Catheters and all instruments used should aseptic.

We have seen more than one case of acute cystitis caused by a filthy catheter. Where you find this trouble engrafted upon a case of chronic gonorrhea, which is often the case, be careful that the urethra is as near free from gonococcus as it is possible to make it before you attempt to irrigate the bladder. The remedies generally used by the writer for this purpose are solutions of permanganate, nitrate of silver or boracic acid according to indications. It will be remembered that most authors on the subject recommend the systematic passing of the steel sound for the cure of chronic gonorrhea. There is no question of its efficacy in certain cases, but the method we have laid down, we believe is applicable in every case, and will usually cure in much shorter time. In reporting this plan a few years ago, we gave the clinical history of several patients treated thereby. And our object at this time is to review the same and include several very important points discovered since that time, that adds much weight to the results

